# Anticipatory postural adjustments during joint action coordination

**DOI:** 10.1038/s41598-019-48758-1

**Published:** 2019-08-23

**Authors:** A. A. Nogueira-Campos, P. M. Hilt, L. Fadiga, C. Veronesi, A. D’Ausilio, T. Pozzo

**Affiliations:** 1 LabNeuro - Laboratory of Cognitive Neurophysiology, Department of Physiology, Institute of Biological Sciences, Federal University of Juiz de Fora (UFJF), Juiz de Fora, Minas Gerais Brazil; 2 IIT@UniFe Center for Translational Neurophysiology of Speech and Communication, Istituto Italiano di Tecnologia, Via Fossato di Mortara, 17-19 Ferrara, Italy; 3 INSERM U1093 - Cognition, Action et Plasticité Sensorimotrice, Université de Bourgogne, 21078 Dijon, France; 4 Department of Biomedical and Specialty Surgical Sciences, Section of Human Physiology, University of Ferrara, Via Fossato di Mortara, 17-19 Ferrara, Italy

**Keywords:** Motor control, Medical research

## Abstract

There is a current claim that humans are able to effortlessly detect others’ hidden mental state by simply observing their movements and transforming the visual input into motor knowledge to predict behaviour. Using a classical paradigm quantifying motor predictions, we tested the role of vision feedback during a reach and load-lifting task performed either alone or with the help of a partner. Wrist flexor and extensor muscle activities were recorded on the supporting hand. Early muscle changes preventing limb instabilities when participants performed the task by themselves revealed the contribution of the visual input in postural anticipation. When the partner performed the unloading, a condition mimicking a split-brain situation, motor prediction followed a pattern evolving along the task course and changing with the integration of successive somatosensory feedback. Our findings demonstrate that during social behaviour, in addition to self-motor representations, individuals cooperate by continuously integrating sensory signals from various sources.

## Introduction

Imagine a waiter using his right hand to lift a glass of wine on a plate that he is holding with his left hand. The success of such a bimanual asymmetric task depends on the capacity of the waiter to counteract the upward perturbation induced by the unloading movement. In such a context, the central nervous system can anticipate movement consequences and produce anticipatory postural adjustments (APAs)^1,2^. APAs consist of using an efferent copy^3^ of the motor command descending towards the lifting hand to prevent the disturbance exerted on the postural hand.

When the two hands hold the plate and the glass, APAs on the postural hand start before the onset of the unloading action. If a reaching phase precedes the lifting, the visual feedback on the reaching adds to the efferent copy to anticipate the unloading. Interestingly, previous investigations have not provided the appropriate experimental context to understand how these two signals contribute to efficient bimanual interactions. Indeed, subjects either bimanually picked up objects with the two hands already positioned on the recording set up^1,4–7^ or initiated the unloading by pressing a button^8^. Furthermore, when a reaching movement was included, the task was performed without visual feedback^9,10^. The first goal of this study was to investigate the role of visual feedback and to verify its potential additional value in the genesis of APAs by introducing a reaching phase preceding the bimanual load-lifting phase.

The investigation of how vision can impact APAs may be essential to the extension of the scope to a joint action scenario^11^, such as where the waiter offers the glass to a guest. While APAs remain essential to the effectiveness of the dyadic interaction, the sole predictive signal is now provided by the visual cues about the guest’s hand trajectory towards the glass. In the next step of the current experiment, we sought to verify whether APAs in a joint action condition might be driven by visual cues even in the absence of any efferent copy signal. Precisely, we aimed to verify if the existing predictive models for the control of the observer’s (here the waiter) own action could anticipate in real time the effect of the guest’s reaching, grasping and lifting movement. Accordingly, APAs are predicted because action observation elicits subthreshold sensorimotor activations analogous to those recruited during action execution^12–14^. Importantly, this sensorimotor recruitment has already shown some degree of anticipation with respect to the ongoing observed action^15^ and has been proposed to be a key asset in allowing the action prediction of others both in the absence of any interaction^16,17^ and in joint action conditions^18^.

**Figure 1:**
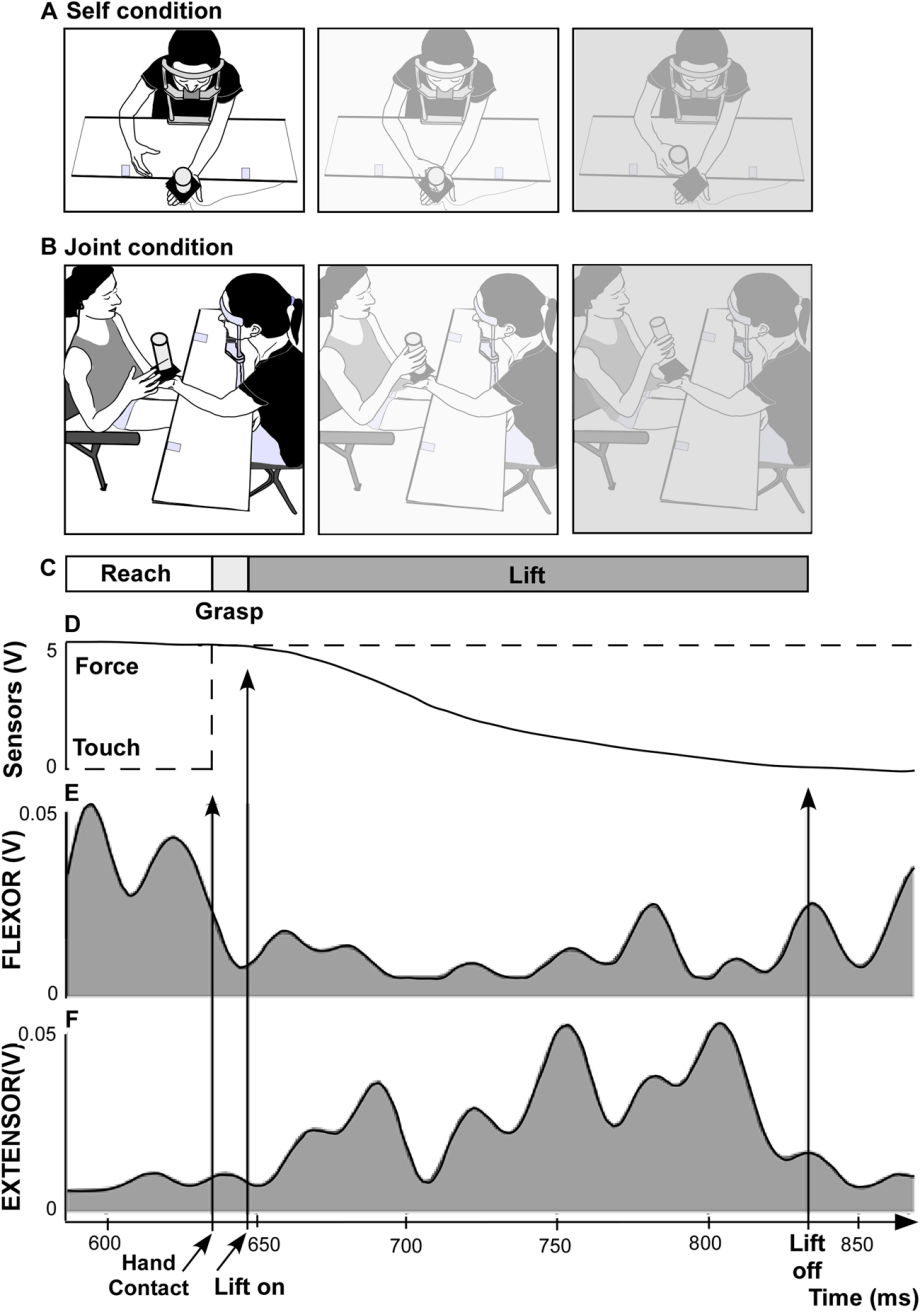
Experimental setup. (**A**) Self condition: frontal view of the carrier holding the object with his left hand and reaching (left), grasping (middle) and lifting (right) the object with his right hand. (**B**) Joint condition: lateral view of the carrier (black dress) holding the object with his left hand, while his partner (gray dress) reached (left), grasped (middle) and lifted (right) with his right hand. In all experimental conditions, the carrier had to keep his left arm flexed on the table with the wrist supinated holding the object in his hand. The bar situated below the pictures (**C**) represents the duration of the different phases of the task: reaching (white), grasping (light grey), lifting (dark grey) for a typical trial (self condition with eyes open). These phases were determined based on touch and load sensors displayed below (**D**). The two lower panels show the muscle activity of wrist flexor (**E**) and extensor (**F**) muscles for the same trial. Vertical lines indicate the moment at which the object was touched (*hand contact*), at which the lifting of the object started (*lift on*) and at which the lifting ended (*lift off*).

The present study achieved these goals by applying a classical APA paradigm to quantify motor prediction while wrist flexor and extensor muscle activities were recorded during a reach-to-grasp load-lifting task performed either alone (self condition) or with the help of a partner (joint condition) (Fig. 1). The task was divided into three movement phases, reaching, grasping and lifting, where each reflects the presence of different combinations of predictive signals, including the efferent copy, visual and somatic signals. Thus, because one of the key tenets of APAs is that motor behaviour must be self-produced^8^, we predicted that APAs would be independent of visual feedback in the self condition, in which the task was executed alone. In this sense, running the task with eyes open or closed should, in principle, produce identical results if visual feedback is not incorporated in the generation of APAs. In the dyadic scenario, APAs need to be implemented to engage safe and efficient joint action coordination. However, the absence of the efferent copy signal puts the burden of anticipation upon a different set of signals. Only somatic (i.e., tactile cues from object-hand haptic interaction and force change during lifting) and visual input (i.e., hand reaching trajectory) may be used in this case. Importantly, somatic cues have far less predictive power than visual cues because they are available only after object contact. Here, the task was also executed with eyes open and closed, so that in one case, both somatic and visual cues were present, while in the other case, only somatic signals were made available.

## Results

Herein, the APAs were recorded during a reach-to-grasp load-lifting task made by the own agent or an interacting dyad. Two conditions were tested: *self*, in which participants (*carriers*) had to reach, grasp and lift the object from a plate maintained in their left arm, and *joint*, in which another participant (*partner*) lifted the object from the plate supported by the carrier who kept his eyes open or closed. Surface electromyography of the wrist flexor and extensors was recorded from the supporting arm (Fig. 1). The results presented below revealed that the muscle adjustments evolved along the task course and were altered by the integration of successive somatosensory feedback. All numerical results presented in this part are expressed as the mean ± SEM.

### Task learning effects

The first analysis considered the difference between the two recording sessions of each condition to evaluate a potential learning effect. No significant difference was found between experimental blocks for the duration of each movement phase. Additionally, APAs for both flexor and extensor aligned with hand contact, and lift on and lift off did not differ among blocks (see Supplementary Material). Although a learning effect has been previously reported when an unloading task was triggered by pressing a button^8^ or by lifting an independent load^19^, no learning effect was observed in the present task. Thus, the following analyses were run on all trials.

### Movement phase duration

**Figure 2:**
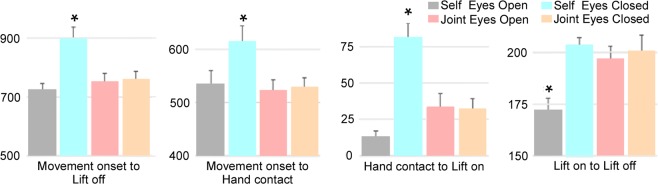
Durations in milliseconds of each movement phase for each experimental condition. From left to right: (1) trial duration: from the onset of the reaching movement to the end of object lifting (lift off); (2) reaching duration: from movement onset to hand contact; (3) grasping duration: from hand contact to the onset of object lifting (lift on); (4) lifting duration: from lift onset (lift on) to lift offset (lift off). Bars represent standard errors. Asterisks show significant differences (p < 0.05) from other conditions.

This analysis was conducted to control for task performance based on time spent to complete the task in each condition and examine the crucial role of visual feedback in task achievement. The duration of the reach and grasp actions (all trial: *movement onset*-*lift off*; reach: *movement onset*-*hand contact*; grasp: *hand contact*-*lift on*) were significantly longer in the self condition with eyes closed (all: 898 ± 40 ms, reach: 613 ± 32 ms, grasp: 81 ± 12 ms) than in the three other conditions (self eyes open: all = 718 ± 28 ms, p < 0.001, reach = 534 ± 26 ms, p < 0.001, grasp = 13 ± 4 ms, p < 0.001; joint eyes open: all = 753 ± 28 ms, p = 0.004, reach = 523 ± 20 ms, p = 0.005, grasp = 33 ± 9 ms, p = 0.01; joint eyes closed: all = 760 ± 28 ms, p = 0.006, reach = 527 ± 19 ms, p = 0.01, grasp = 32 ± 7 ms, p = 0.01) (Figs 1 and 2 in the Supplementary Material). Thus, reaching duration was comparable when performed by the partner or the carrier herself. This observation excluded any mechanical effect due to potential higher hand momentum on the consecutive grasping and unloading phases and made possible a suitable comparison of APAs between the self eyes open and joint conditions (eyes open and eyes closed). In contrast, the duration of the lifting phase (*lift on*-*lift off*) was shorter in the self eyes open condition (172 ± 6 ms) than in the other conditions (self eyes closed: 204 ± 4 ms, p = 0.001; joint eyes open: 197 ± 7 ms, p = 0.01; joint eyes closed: 201 ± 8 ms, p = 0.01). No other significant difference was found. See Figs 2–4 in the Supplementary Material for data distribution across trials.

**Figure 3:**
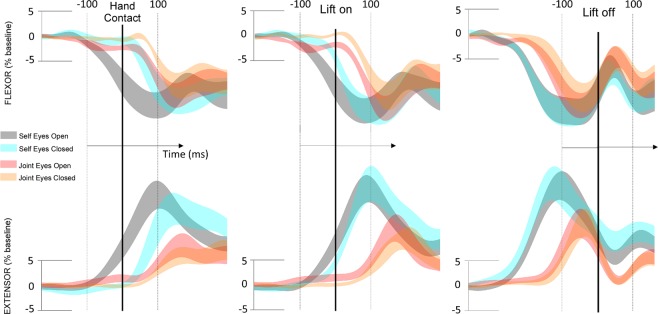
Standard errors around the mean of electromyography activity of flexor (upper panels) and extensor (lower panels) muscles, aligned at time of contact with the object (*hand contact*), lift onset (*lift on*) and lift offset (*lift off*), for each experimental condition.

**Figure 4:**
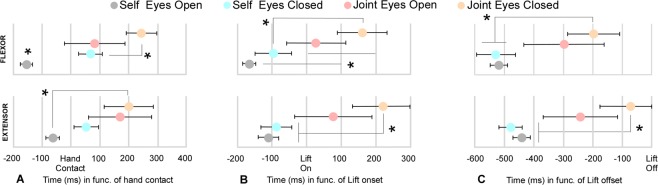
EMG flexor deactivation and extensor activation onset as a function of the time until the hand comes into contact with the object (*hand contact*, left panel, A), the lift onset (middle panel, B) and the lift offset (right panel, C). Asterisks show significant differences (p < 0.05) from other conditions.

### EMG activation/deactivation onset

The wrist flexor and extensor muscle activity was computed by each experimental condition to evaluate the effect of efferent copy, visual and somatic signals on the anticipatory adjustments. More specifically, the self conditions (eyes open and eyes closed) could be used to discern the effect of visual feedback on APA before lifting. The joint condition with eyes open closely matches the information present in the self eyes open condition but lacking the critical contribution of efferent copy signals. However, the joint with eyes closed condition lacks both visual feedback and efferent copy while keeping only tactile and force feedback. Figure 3 shows the average flexor deactivation and extensor activation time course with respect to each of the three identified time points (*hand contact*, *lift on* and *lift off*). Figure 4 illustrates the muscle deactivation (flexor) or activation (extensor) onset related to these same points. See also Fig. 5 of Supplementary Material.

**Figure 5:**
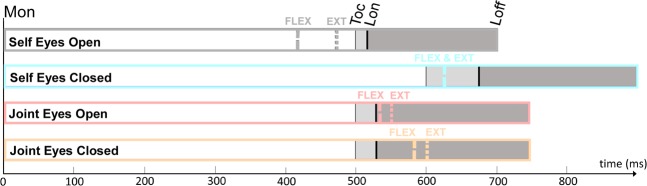
Illustration of the averaged onset of flexor (FLEX) deactivation and extensor (EXT) activation as a function of the defined phases of the task. The illustration depicts the time point at which the muscle adjustment computed in Fig. 3 started along the task for each experimental condition. Movement onset (*Mon*), hand contact (*Toc*), lift onset (*Lon*), and lift off (*Loff*).

### APA before time of hand contact

Flexor onset deactivation appeared significantly sooner in the self eyes open condition (−78 ± 11 ms) than in the three other conditions (self eyes closed: 34 ± 21 ms, p = 0.0001, t = −5.7; joint eyes open: 41 ± 52 ms, p = 0.02, t = −2.2; joint eyes closed: 122 ± 26 ms, p = 0.0001, t = −6.7). Additionally, there was a significant difference between the self eyes closed and joint eyes closed conditions (p = 0.03, t = −2.7). Extensor activation in the self eyes open (−32 ± 12 ms) condition occurred significantly earlier than in the joint eyes closed condition (100 ± 43 ms; p = 0.002, t = −3.6). No other significant differences were found (Fig. 4A).

### APA before lift onset

Although the interval between time of hand contact with the object and the onset of its lifting was short, we also computed the latencies related to this time point. Flexor deactivation in the self eyes open condition (−84 ± 10 ms) occurred significantly sooner than that in the joint conditions (joint eyes open: 13 ± 43 ms, p = 0.05, t = −2.4; joint eyes closed: 80 ± 36 ms, p = 0.001, t = −4.8). Flexor deactivation during the joint eyes closed condition was also significantly later than that during the self eyes closed condition (−49 ± 26 ms, p = 0.02, t = −2.4). Extensor activation occurred significantly sooner in the self conditions (self eyes open: −55 ± 14 ms, p = 0.001, t = −4.2; self eyes closed: −43 ± 23 ms, p = 0.01, t = −3.1) than in the joint eyes closed condition (111 ± 45 ms) but did not differ from that in the joint eyes open condition (39 ± 56 ms, p = 0.21, t = −1.4; Fig. 4B).

The latencies shown herein can be assumed to be in agreement with those previously reported for proximal muscles, i.e., biceps brachii (−31 ms) and brachioradialis (−15.5 ms), preceding the onset of unloading^2,4,10,19^. However, these studies did not control for the contribution of visual information. Another study described the onset of flexor muscle anticipation to be approximately 100 ms before a ball impact over the hand in a visually oriented task^20^. When the extensor was investigated, a later activation was observed^4,9,10^ as shown herein.

### APA before lift offset

The onset of flexor deactivation and extensor activation in the self eyes open (flexor: −259 ± 15 ms; p = 0.001, t = −3.7; extensor: −221 ± 15 ms, p = 0.007, t = −3.6) and self eyes closed condition (flexor: −264 ± 33 ms, p = 0.03, t = −2.8; extensor: −239 ± 20 ms, p = 0.007, t = −4.1) appeared significantly sooner than that in the joint eyes closed condition (flexor: −98 ± 44 ms, extensor: −37 ± 52 ms). However, there was no difference in onset between the self conditions and the joint eyes open for either muscle (flexor: −148 ± 68 ms, p = 0.11, t = −1.9; extensor: −121 ± 63 ms; p = 0.14, t = −2.0; Fig. 4C).

To better visualize these results, Fig. 5 illustrates the averaged onset of flexor deactivation and extensor activation as a function of movement phases and experimental conditions. The figure highlights a clear effect of efferent copy and visual information on the timing of APAs, as shown for the self eyes open condition in Fig. 3. In the self eyes closed condition, APAs started after *hand contact* (Toc), and the grasping duration was prolonged. Finally, delayed APA was observed in the joint conditions, especially with eyes closed.

## Discussion

Herein, we analysed APAs using a reach-to-grasp load-lifting task performed by a single agent or an interacting dyad, with or without visual feedback. When subjects performed the task by themselves, APAs were observed before the *hand contact*, reflecting predictive control of the dynamic disturbance of the reaching movement onto the supporting hand. Without visual input, these early bimanual APAs were delayed until the grasping onset. Importantly, in the joint action scenario, APAs were exclusively recorded when tactile and force feedback signals were available. These results are discussed below considering the predictive function of visual perception when the task is performed in bimanual and social contexts.

In the self condition with eyes open, the APA investigation revealed clear forearm muscle changes and flexor deactivation starting approximately 80 ms before the hand touched the object (referring to reaching APAs). This result is difficult to compare with previous investigations where the two hands systematically gripped on the object to be lifted^1,4,5,19^, not allowing for the verification of the presence of APAs during the reaching phase. In this study, the load-bearing hand was not mechanically stabilized, and participants were not asked to stabilize it. Such a free postural context introduced large spatial and temporal uncertainties in the bimanual coordination. Early reaching APAs likely contribute to the precise synchronization of the two subtasks under visual control, minimization of the mechanical perturbation of the moving hand towards the supporting hand, and finally to the smooth lifting of the object. An oculomotor saccade towards the object ahead of the hand-grasping phase^21^ combined with the efferent copy signal of the command to the lifting hand could provide crucial inputs in producing reaching APAs. Indeed, APAs during arm-pointing towards a target from a standing position have been shown to depend on oculomotor timing^22^, a result suggesting that the commands to eye and postural muscles are created at the same time. Without visual cues, flexor deactivation onset was systematically recorded after grasping (referring to grasping APAs), which is when haptic input (from the receptors in muscles, joints and skin) can combine with the efferent copy signal for the coordination of the two hands.

In the dyadic context, anticipated muscle changes were recorded after grasping onset. Because the presence of APAs has never been investigated in the presence of only visual feedback, a comparison with other findings^4,23–25^ is difficult. The anticipatory control of load impact recorded during ball catching^20^ is of the same category; however, a continuously accelerating movement of a non-living object is more predictable than an accelerating-decelerating hand movement performed by a human.

Nonetheless, even delayed flexor deactivation and extensor activation recorded before the lift off (referring to lifting APAs) can compensate for the force generated by the opposite hand and produce efficient postural adjustments. During a classical bimanual load-lifting task, APAs were recorded approximately 30–20 ms before the lift off^1^ or sooner^7^. The duration of the present haptic interactions (including the grasping and lifting phases) is thus compatible with the timing of sensorimotor loops engaged in corrective actions (~100 ms)^21^. A fast cutaneous response (approximately 50 ms)^26^ and a modulation of the flexor deactivation of the supporting hand (approximately 100 ms before the lift off in the joint eyes closed condition) to assist the lifting movement are still possible when visual input and efferent copy signals are lacking. Subsequently, a longer lifting phase recorded in the dyadic condition creates the temporal condition for a sensorimotor dialogue between the dyad, where the load-bearing hand assists the lifting hand. This dialogue, exclusively observable when the task is performed without any device or instruction artificially stabilizing the two hands, creates the temporal condition for controlling the task in the most insecure context, supporting the hypothesis that APAs play a dynamic role in postural transition and provide additional force for task goal achievement^27–30^.

Notwithstanding, following the common hypothesis that observed action is simulated with one’s own motor repertoire^31,32^ in addition to behavioural data showing that perception and action planning are coded in a common representational medium^33–35^, we predicted that we would observe early grasping APAs in both dyadic and bimanual conditions. Specifically, the visual observation of others’ actions has been demonstrated to recruit both the motor^36–38^ and the somatic system^39–41^. These activations have been reported to anticipate the temporal deployment of observed actions^42^. More recently, behavioural findings obtained in a social context proposed that the reuse of one’s own bimanual model could have positive effects on the prediction of the action timing of a co-actor^18^. Conversely, our results suggest that the observer’s and actor’s internal models did not fully overlap. Accordingly, APAs were only present as soon as visual input had been combined with tactile and force feedback. Thus, the visual cues from the partner’s action did not provide information to completely predict the dynamic disturbance to occur during the interaction, at least in the context of the unloading task tested here. However, an anticipation was observed in the dyadic condition with eyes open compared to that with eyes closed (Fig. 5).

Several causes could limit the predictive function of visual perception of actions of two interacting agents and thus promote a gradual sensorimotor integration to improve social interactions. Hand reaching movements, even if less variable as when performed synchronously and without physical interaction^43^, remain strongly subject dependent and much less predictable than non-living object kinematics^44,45^. Furthermore, self-bimanual movements represent a special case of multitasking requiring the organization of multiple command streams to control two effectors in addition to their temporal sequencing. According to Wiesendanger & Serrien^46^, the dyadic condition mimics a split-brain situation where the corollary discharge of the motor command to the reaching hand can no longer be relayed to subcortical structures that modulate the commands to the postural hand. Thus, a considerable amount of neural activity related to the ipsilateral limb available in the self condition^47–49^ is missing in the joint condition. Specifically, the basal ganglia^50^ and the cerebellum^51^ modulate hemispheric interactions during bimanual tasks. Investigations performed in patients with callosal lesions have shown similar desynchronization of two interacting hands in vision and no-vision conditions. These results indicate the major role of the corpus callosum in exchanging sensory information about left and right limb motions and of the basal ganglia in adjusting the postural and the moving hand^52,53^. Furthermore, a previous artificial split-brain experiment revealed that visual guidance alone was insufficient for perfect coordination of two independent arms^54^. Finally, the specificity of the task tested here can partly explain the delayed APAs in a dyadic context. Indeed, we did not test the effect of the object weight or the role of the waiter’s hand posture (e.g., either holding by the top or the middle of the object) on the physical interaction. In the case of a heavier object requiring greater mechanical compensation on the load-bearing hand, action observation could help in producing early APAs.

In summary, our results show that visual perception of action and associated motor resonance do not completely help internal variable adjustment during a classical load-lifting task performed by two agents. Rather, our findings demonstrate that in addition to self-motor representations, individuals adapt real-time cooperation by continuously integrating sensory signals from various sources.

## Methods

### Participants

Seventeen couples of individuals took part in the experiment (8 man-man and 9 woman-woman; mean age: 25.5 ± 2.5 SD). All participants had normal sensory motor abilities and did not present any neurological or psychiatric disorders. No explicit information was given about the purpose of the study before the experiment. All participants gave informed consent to participate in the experiment. All procedures were approved by the local Ethics Committee, Comitato Etico della Provincia di Ferrara (approval No. 170592) and complied with the Declaration of Helsinki as of 2008.

### Experimental procedure

The two participants sat comfortably on chairs positioned face-to-face separated by a table (dimension: 1 × 0.3 m; Fig. 1). In each couple, one participant was designated as the “carrier”, and the other as the “partner”. Roles were kept the same during the entire experiment. The carrier held stable an object positioned on his left hand until this object was lifted. The object was a touch-sensitive cylinder weighing 300 g (6 × 18 cm; diameter × height). The carrier held the object on a flat tray fixed to his hand by means of a Velcro strap. The tray was made of two platforms spaced 3 cm (dimension: 10 × 10 the top wood and 7 × 7 cm the bottom one) to fit four load cells between them. The left arm of the carrier was kept flexed on the table with the wrist supinated and fingers pointing forward in an unconstrained posture throughout the entire experiment session. Additionally, the carrier’s head was kept stable on a chinrest placed on the table in front of him. This device allowed a constant position throughout the experiment.

**Figure 6:**
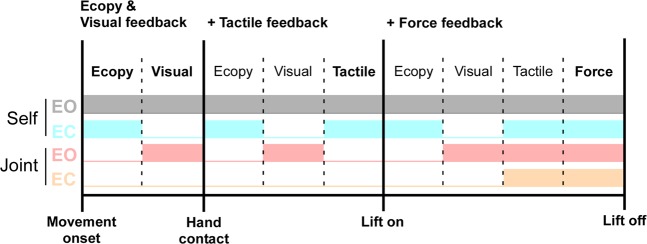
General schema showing the signals available for prediction in each of the three phases. *Start*: for reaching movement onset; *hand contact*: time of finger contact with the object; lift onset (*lift on*), lift offset (*lift off*). In the self condition, the carrier could use efferent copy signals from both hands, visual signals from the reaching hand and somatic information from the supporting hand, in addition to the force changes associated with the interaction during bimanual coordination. In contrast, in the joint conditions, only visual input (i.e., hand reaching trajectory) and somatic signals (i.e., tactile cues from object-hand haptic interaction and force change) during the coordination of the dyad may be used. In addition, the joint eyes closed condition lacks visual input. Shades of grey, blue, red and orange represent available information in each phase for the self eyes open (EO), self eyes closed (EC), joint eyes open and joint eyes closed conditions, respectively. Efferent copy (ECopy), visual, tactile or force feedback signals become progressively available during the task.

In the first experimental condition, the carrier performed the same task by her/himself (self condition; Fig. 1A) by holding the tray with his left hand while reaching, grasping and lifting the object with her/his right hand. In a second experimental condition, the partner had to reach, grasp and lift the carrier’s object with his right hand (joint condition; Fig. 1B). These two conditions were carried out with the carrier having either the eyes open (EO) or closed (EC). In all conditions, reaching movement onset was self-paced and detected by a touch sensor fixed on a square plate (side: 10 cm), marking on the table the starting position of the partner’s right hand. The combination of these experimental conditions allowed us to evaluate the significance of efferent copy, visual and somatic signals on an agent’s capability to anticipate object lifting (Fig. 6). Thus, in the self condition, the carrier could use efferent copy signals from both hands, visual signals from the reaching hand and somatic information of the supporting hand, in addition to the force change from the interaction during bimanual coordination. In contrast, in the joint condition, only visual input (i.e., hand reaching trajectory) and somatic signals (i.e., tactile cues from the object-hand haptic interaction and force change) during the coordination of the dyad may be used.

In fact, to verify the effect of integrating somatic and visual inputs with the efferent copy on APAs, three movement phases were identified, each reflecting the presence of different combinations of predictive signals. These phases are aligned to the onset of the hand touching the object (*hand contact*), lift onset (*lift on*) and lift offset (*lift off*). As illustrated in Fig. 6, efferent copy and visual feedback (visual fb) are progressively integrated with tactile feedback (Tfb) and force feedback (Ffb) in the self condition with eyes open, while the self with eyes closed condition lacks visual feedback. These conditions helped to discern the effect of visual feedback on APA before lifting. In contrast, the joint condition with eyes open closely matches the information present in the self eyes open condition, though lacking the critical contribution of efferent copy signals. Finally, the joint with eyes closed condition lacks both visual feedback and efferent copy, while keeping only tactile and force feedback. Thus, this condition was used as a control to measure APAs when both efferent copy and visual feedback signals were lacking.

The experiment was completed in eight blocks with two of each experimental condition: the self condition with eyes open, the self condition with eyes closed, the joint condition with eyes open or the joint condition with eyes closed. The order of blocks and conditions was randomized for each participant. Each block consisted of 20 trials and was followed by 5 min of rest. During rest periods, instructions concerning the upcoming block were given. Before the recording was begun, a variable number of training trials (~8 trials with eyes open and eyes closed in both conditions) were run until the participants felt confident with the task. The entire procedure lasted approximately 40 min.

### Electromyographic and behavioural signal acquisition

All data were acquired via an acquisition board (CED Power1401-3A, Cambridge Electronic Design, Cambridge, UK) and stored on a PC with Dasylab Software (MCC corporate, Norton, USA).

The electromyographic (EMG) signal was recorded using a wireless system (Aurion, Italy) amplifying (gain of 1,000) and digitizing the data at 2000 Hz. Electrodes were arranged according to a bipolar tendon-belly montage over the *flexor digitorium superficialis* (flexor; Fig. 1E) and the *extensor digitorium communis* (extensor; Fig. 1F) of the carrier’s left arm for all conditions. Three other types of behavioural data were simultaneously acquired: (1) the touch signal coming from the right hand starting place (binary signal: value 5 if the hand is in contact with the starting place, value 0 from the start of the reaching movement); (2) touch signal coming from the object held by the carrier (binary signal: value 5 when the hand is in contact with the object, value 0 before the grasp of the bottle by the right hand; Fig. 1D), and (3) weight-related signal coming from the four load cells situated in the tray (continuous signal; Fig. 1D). These signals were recorded to define the movement phases and the precise events of the release of the object from the tray supported by the carriers.

### Data analysis

#### Definition of movement phases

The right-hand movement onset (*Mon*) was determined as the first point at which the touch signal coming from the starting place reached a null value (for a minimum of 50 ms). The right-hand time of contact with the object (*hand contact*) was determined as the first point at which the touch signal coming from the object reached a value of 5 (for a minimum of 50 ms; Fig. 1D). The beginning and the end of the lifting phase (respectively *lift on* and *lift off*) were extracted from the tray’s load signal. *Lift on* was defined as the first time point dropping below 95% of the maximal load value (for a minimal duration of 50 ms). *Lift off* was defined as the first time point dropping below 5% of the maximal load value (for a minimal duration of 50 ms).

Using these time points, the duration of each movement phase was computed (Fig. 1C): (1) trial duration: from right-hand movement onset to *lift off*; (2) reaching duration: from right-hand movement onset to *hand contact*; (3) grasping duration: from *hand contact* to *lift on*; (4) lifting duration: from *lift on* to *lift off*.

#### EMG processing

EMG signals of each muscle were first visually inspected trial-by-trial to control for the presence of recording artefacts. No trial was discarded after this procedure. Flexor and extensor EMGs for each trial were first high-pass filtered (20 Hz) and then digitally full-wave rectified and low-pass filtered (Butterworth filter, cut-off frequency of 10 Hz, zero-phase distortion)^55^ and normalized to 1,000 time steps. Compared to the tonic activity enabling the maintenance of the object on the tray, the unloading was compensated via an increase in extensor and a decrease in flexor activity (Fig. 1E,F). To evaluate these modulations, EMG signals were cut and temporally aligned to *hand contact* (from *Hand contact* −500 ms to *hand contact* +1000 ms), *lift on* (from *lift on* −500 ms to *lift on* +1000 ms), and *lift off* (from *lift off* −650 ms to *lift off* +850 ms) for each trial. After a visual inspection, we determined the onset on average curve for each condition and each subject, to avoid extracting local minima linked to recording noises. To compute this average curve, we firstly aligned all trials relatively to an objective external event (hand contact, lift onset and lift offset). For each alignment, each subject and each experimental condition, we computed the mean activity of flexor and extensor muscles. We then evaluated the presence of extensor activations and flexor deactivations using a semi-automatic algorithm. For each subject, we defined the onset of activation (extensor) or deactivation (flexor) as the first time point at which muscle activity was higher (extensor) or lower (flexor) than the tonic baseline activity for a minimum duration of 150 ms. Baseline activity was computed for each subject and each muscle as the mean muscle activity on a 350-ms window (from *hand contact* −550 ms to *hand contact* −200 ms) adding (extensor) or subtracting (flexor) 2 standard deviations.

### Statistical analysis

The Shapiro-Wilk test was used to check the normality assumption for parametric tests. Data were not all normally distributed (p < 0.05). Thus, all statistical comparisons were performed using two-tailed permutation tests (5000 permutations; MATLAB function mult_comp_perm_t1). All P-values were corrected for multiple comparisons using the Benjamini-Hochberg False Discovery Rate (MATLAB function fdr_bh).

## Supplementary information

Supplementary material

## Data Availability

The authors confirm that all data supporting the findings of this study are available within this manuscript and its supplementary materials. However, the raw data are also available from the corresponding author upon request.
