# Publisher Correction: Anticipatory postural adjustments during joint action coordination

**DOI:** 10.1038/s41598-019-55184-w

**Published:** 2019-12-04

**Authors:** A. A. Nogueira-Campos, P. M. Hilt, L. Fadiga, C. Veronesi, A. D’Ausilio, T. Pozzo

**Affiliations:** 1 LabNeuro - Laboratory of Cognitive Neurophysiology, Department of Physiology, Institute of Biological Sciences, Federal University of Juiz de Fora (UFJF), Juiz de Fora, Minas Gerais Brazil; 2 IIT@UniFe Center for Translational Neurophysiology of Speech and Communication, Istituto Italiano di Tecnologia, Via Fossato di Mortara, 17-19, Ferrara, Italy; 3 INSERM U1093 - Cognition, Action et Plasticité Sensorimotrice, Université de Bourgogne, 21078 Dijon, France; 4 Department of Biomedical and Specialty Surgical Sciences, Section of Human Physiology, University of Ferrara, Via Fossato di Mortara, 17-19, Ferrara, Italy

Correction to: *Scientific Reports* 10.1038/s41598-019-48758-1, published online 23 August 2019

The original PDF version of this Article contained incorrect A-F labels in Figure 1A and 1B. This has now been corrected in the PDF; the HTML version of the paper was correct from the time of publication.

