# Supplementary material for: Anticipatory postural adjustments during joint action coordination
Source: Sci Rep. 2019 Aug 23;9:12328. doi: 10.1038/s41598-019-48758-1 (PMC6707290; doi:10.1038/s41598-019-48758-1)
Supplement: Supplementary file 1 — Supplementary material [file 41598_2019_48758_MOESM1_ESM.pdf]

Anticipatory postural adjustments during joint action coordination

Nogueira-Campos AA[1*], Hilt PM[2*], Fadiga L[2,4], Veronesi C[4], D'Ausilio A[2,4] , Pozzo T[2,3]

[1] LabNeuro - Laboratory of Cognitive Neurophysiology, Department of Physiology, Federal University of Juiz de Fora (UFJF), Juiz de Fora, Minas Gerais, Brazil.

[2] IIT@UniFe Center for Translational Neurophysiology of Speech and Communication, Istituto Italiano di Tecnologia, Via Fossato di Mortara, 17-19, Ferrara, Italy.

[3] INSERM U1093 - Cognition, Action et Plasticité Sensorimotrice, Université de Bourgogne, 21078, Dijon, France.

[4] Department of Biomedical and Specialty Surgical Sciences, Section of Human Physiology, University of Ferrara, Via Fossato di Mortara, 17-19, Ferrara, Italy.

Supplementary material

FLEXOR – onset of deactivation

- Data (mean ±ste)

	Hand contact		Lift on		Lift off	
	**Block 1**	**Block 2**	**Block 1**	**Block 2**	**Block 1**	**Block 2**
**Self eyes open**	-51 ±13	-101 ±31	-71 ±11	-99 ±30	-194 ±51	-236 ±14
**Self eyes closed**	32 ±46	35 ±16	-41 ±45	-24 ±33	-213 ±53	-230 ±29
**Joint eyes open**	7 ±47	58 ±39	-1 ±45	-59 ±53	-120 ±57	-137 ±51
**Joint eyes closed**	129 ±65	127 ±22	113 ±66	112 ±42	-61 ±75	-78 ±48

- Statistics (permutation test): Block 1 vs Block 2

	Hand contact	Lift on	Lift off
**Self eyes open**	p=0.44, t=1.50	p=0.83, t=0.79	p=0.82, t=0.85
**Self eyes closed**	p=0.96, t=-0.07	p=0.86, t=-0.34	p=0.82, t=0.50
**Joint eyes open**	p=0.95, t=-0.77	p=0.71, t=-1.15	p=0.82, t=0.26
**Joint eyes closed**	p=0.96, t=0.04	p=0.99, t=0.02	p=0.88, t=0.17

EXTENSOR – onset of activation

- Data (mean±ste)

	Hand contact		Lift on		Lift off	
	**Block 1**	**Block 2**	**Block 1**	**Block 2**	**Block 1**	**Block 2**
**Self eyes open**	-8 ±23	-32 ±12	-15 ±22	-44 ±13	-139 ±55	-216 ±13
**Self eyes closed**	52 ±11	-55 ±50	-10 ±8	-115 ±46	-215 ±10	-299 ±38
**Joint eyes open**	-18 ±74	48 ±56	-48 ±71	34 ±64	-110 ±65	-158 ±59
**Joint eyes closed**	118 ±63	112 ±63	118 ±63	87 ±60	-110 ±71	-49 ±62

- Statistics (permutation test): Block 1 vs Block 2

	Hand contact	Lift on	Lift off
**Self eyes open**	p=0.44, t=1.34	p=0.48, t=1.49	p=0.22, t=1.55
**Self eyes closed**	p=0.39, t=2.06	p=0.36, t=2.17	p=0.22, t=2.09
**Joint eyes open**	p=0.73, t=-1.09	p=0.71, t=-1.02	p=0.82, t=0.65
**Joint eyes closed**	p=0.96, t=0.08	p=0.83, t=0.46	p=0.82, t=-0.75

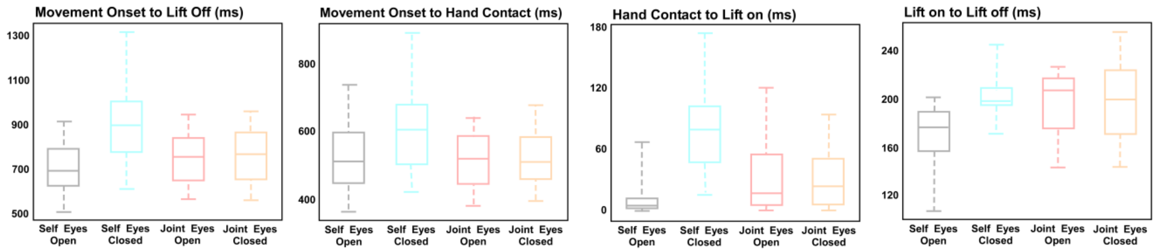

Figure 1. Box-and-whisker plots for duration in milliseconds (ms) of each movement phase. From left to right: (1) trial duration: from the onset of reaching movement to the end of object lifting (lift off), (2) reaching duration: from movement onset to hand contact, (3) grasping duration: from hand contact to the onset of the object lifting (lift on), (4) lifting duration - from lift onset (lift on) to lift offset (lift off).

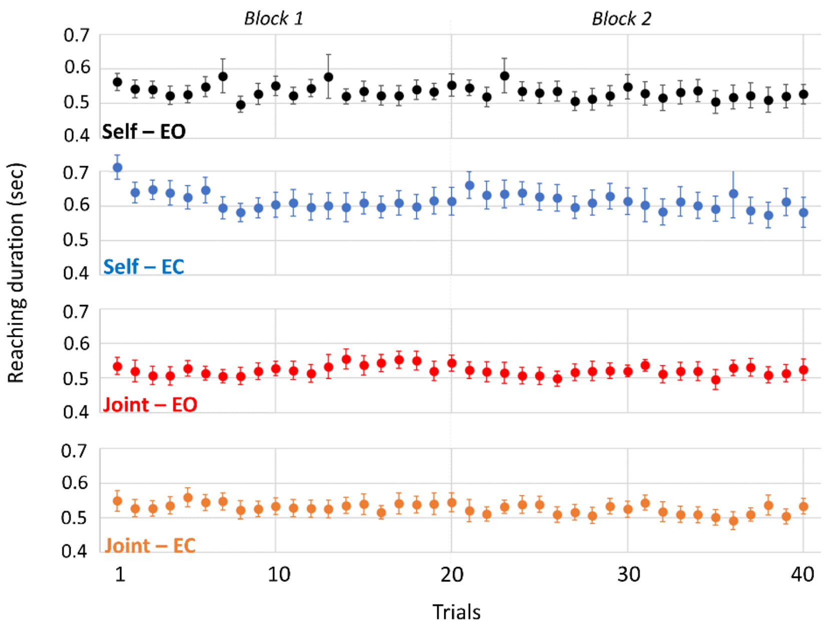

Figure 2: Data distribution of reaching duration in seconds across recording sessions.

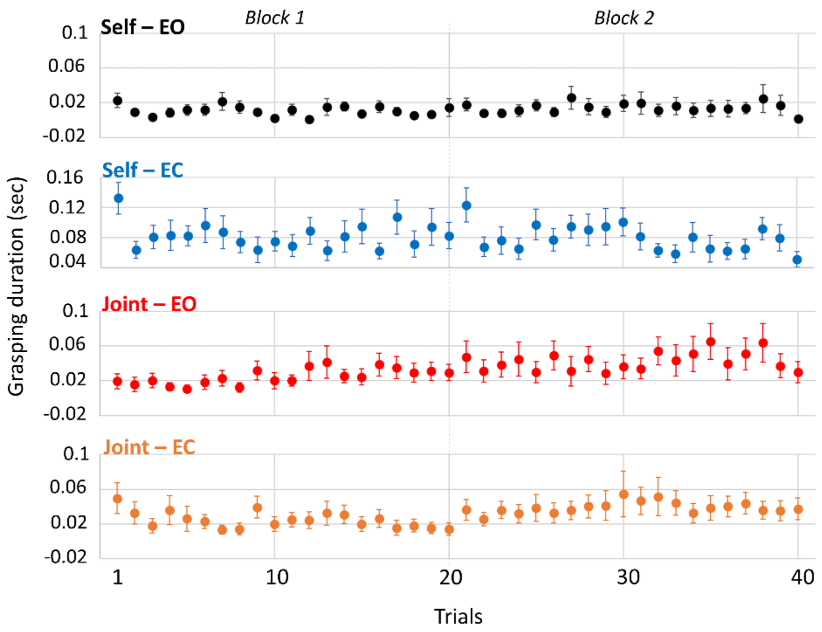

Figure 3: Data distribution of grasping duration in seconds across recording sessions.

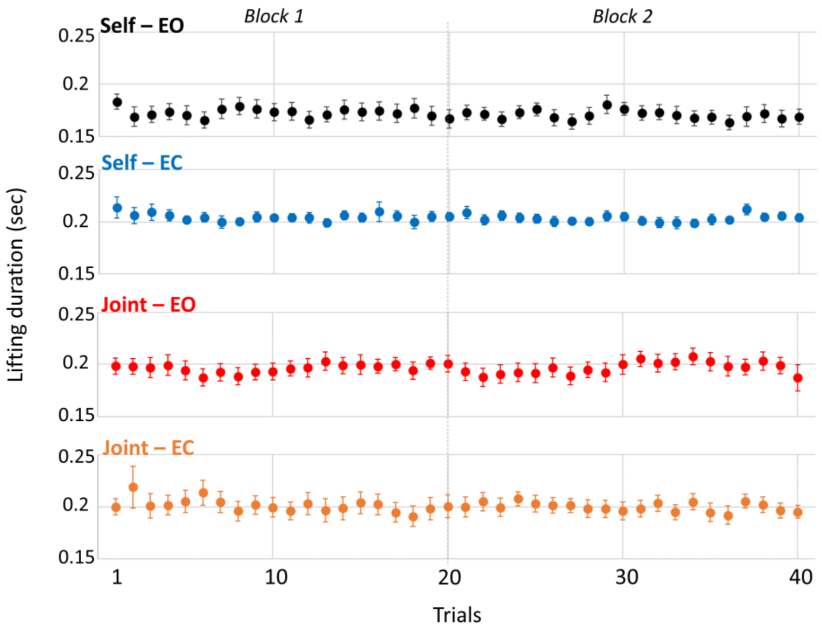

Figure 4: Data distribution of lifting duration in seconds across recording sessions.

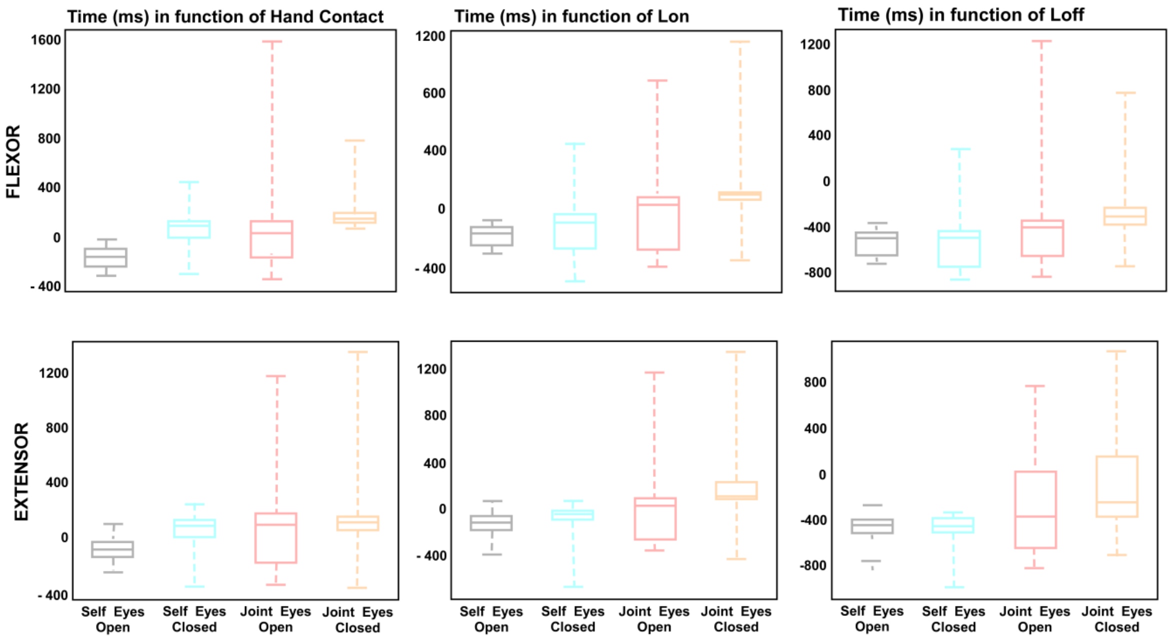

Figure 5. Box-and-whisker plots for electromyography activity of flexor (upper panels) and extensor (lower panels) muscles, aligned on time of contact with the object (*hand contact*), lift onset (*lift on*) and lift offset (*lift off*), for each experimental condition.
